# Osteosarcoma inheritance in two families of Scottish deerhounds

**DOI:** 10.1186/s40575-017-0042-8

**Published:** 2017-03-17

**Authors:** John E. Dillberger, Sara Ann McAtee

**Affiliations:** P.O. Box 2118, Nashville, IN 47448 USA

**Keywords:** Osteosarcoma, Scottish deerhound, Neoplasia, Inheritance, Genetic variant

## Abstract

**Background:**

Osteosarcoma is the most common neoplastic disease in Scottish Deerhounds. For Deerhounds, a 2007 population-based study concluded that a single dominant genetic factor largely governed disease risk. For Greyhounds, Rottweilers, and Irish Wolfhounds, a 2013 genome-wide association study found multiple genetic markers in each breed, with each marker only weakly associated with the disease.

We obtained from two breeders the pedigrees, age (if alive) or age at death, and osteosarcoma status for two families of Scottish Deerhounds, designated Cohorts K and T. A dog was considered unaffected only if it was osteosarcoma-free and at least 8.5 years old. We analyzed the data in two ways, by assuming either a single recessive genetic factor or a single dominant genetic factor with high penetrance.

**Results:**

Cohort K contained 54 evaluable dogs representing 12 litters. Cohort T contained 56 evaluable dogs representing eight litters. Osteosarcoma seemed clearly heritable in both cohorts; however, having a parent with osteosarcoma raised a pup’s risk of developing osteosarcoma to 38% for Cohort K but 78% for Cohort T, suggesting the possibility of different genetic risk factors in each cohort. In Cohort K, osteosarcoma inheritance fit well with a single, recessive, autosomal risk factor, although we could not rule out the possibility of a single dominant risk factor with incomplete penetrance. In Cohort T, inheritance could be explained well by a single, dominant, autosomal risk factor but was inconsistent with recessive expression.

**Conclusions:**

Inheritance of osteosarcoma in two Scottish Deerhound families could be explained well by a single genetic risk factor residing on an autosome, consistent with a 2007 report. In one family, inheritance was consistent with dominant expression, as previously reported. In the other family, inheritance fit better with recessive expression, although the possibility of a dominant genetic factor influenced by one or more other genetic factors could not be ruled out. In either case, the results suggest that there may be at least two different genetic risk factors for osteosarcoma in Deerhounds.

## Plain English Summary

Osteosarcoma (bone cancer) is the most common cancer in Scottish Deerhounds. For Deerhounds, a 2007 study concluded that a single dominant genetic factor largely governed disease risk. For Greyhounds, Rottweilers, and Irish Wolfhounds, a 2013 study found multiple genetic markers in each breed, with each marker only weakly associated with the disease.

We obtained from two breeders the pedigrees, age (if alive) or age at death, and bone cancer status for two families of Scottish Deerhounds, designated Cohorts K and T. A dog was considered unaffected only if it was free of bone cancer and at least 8.5 years old. We analyzed the data in two ways, by assuming either a single recessive genetic factor or a single dominant genetic factor.

Cohort K contained 54 evaluable dogs representing 12 litters. Cohort T contained 56 evaluable dogs representing eight litters. Bone cancer seemed clearly heritable in both cohorts; however, having a parent with bone cancer raised a pup’s risk of developing bone cancer itself to 38% for Cohort K but 78% for Cohort T, suggesting the possibility of different genetic risk factors in each cohort. In Cohort K, bone cancer inheritance fit well with a single recessive risk factor, although we could not rule out the possibility of a single dominant risk factor. In Cohort T, inheritance could be explained well by a single dominant risk factor but was inconsistent with recessive expression.

Inheritance of bone cancer in two Scottish Deerhound families could be explained well by a single genetic risk factor, consistent with a 2007 report. In one family, inheritance was consistent with dominant expression, as previously reported. In the other family, inheritance fit better with recessive expression, although the possibility of a dominant genetic factor influenced by one or more other genetic factors could not be ruled out. In either case, the results suggest that there may be at least two different genetic risk factors for bone cancer in Deerhounds.

## Background

The Scottish Deerhound is a giant breed of dog originally used to hunt Red Deer (Cervus elaphus) in Scotland. According to the breed standard, a Deerhound “should resemble a rough-coated Greyhound of larger size and bone.” The standard recognizes a clear sexual dimorphism in the breed, with males averaging 85 to 110 lb and standing 30 to 32 in. high at the withers, and bitches weighing 75 to 95 lb and standing at least 28 in. high at the withers. The Deerhound’s balance of large size with speed and stamina was necessary to give them some chance of success against their quarry, as a male Red Deer averages 350 to 530 lb.

Scottish Deerhounds as a breed were first recognized by the American Kennel Club (AKC) in 1886, but historical descriptions and illustrations go back centuries earlier. Despite this long history, Deerhounds have always been, and are today, a rare breed. For example, in 2008, the AKC registered only 153 new Deerhounds. By 2013 the AKC ranked the Deerhound by registrations as 166th in popularity out of 178 breeds.

For at least 50 years, the scientific community has understood that large- and giant-breed dogs are at greater risk of osteosarcoma than other breeds [1]. Scottish Deerhounds are no exception. In fact, osteosarcoma is by far the most common neoplastic disease in the breed and one of the leading causes of morbidity and mortality. In 1996 and 2011, one of the authors of this paper (JED) helped the Scottish Deerhound Club of America design, conduct, and analyze the results from health surveys sent to Deerhound owners and breeders. The 2011 survey yielded information on 588 dogs (273 males and 315 bitches). In that population, 24 males and 35 bitches had developed osteosarcoma, representing incidences of 9% and 11%, respectively [2]. In fact, osteosarcoma was the most commonly reported health problem in bitches and the second most commonly reported health problem in males, where it was only slightly less common than heart disease. These results are almost identical to those obtained from the 1996 survey [3].

Because osteosarcoma is often fatal, it is a leading cause of death for Scottish Deerhounds. Of the 588 dogs in the 2011 health survey, 306 were deceased, and the cause of death was reported for 274 of them (122 males and 152 bitches). Osteosarcoma was the single most common cause of death in each sex, accounting for 22% of the mortality in bitches and 16% of the mortality in males [4].

Osteosarcoma risk is heritable in Scottish Deerhounds. A 2007 study [5] used pedigrees and phenotype information provided by owners and breeders for a population of over 1,200 related Deerhounds to model the inheritance of the osteosarcoma phenotype. The model was then queried using variance component analysis to estimate the heritability, and segregation analysis to infer the presumptive mode of inheritance. Heritability was estimated to be 0.69. Osteosarcoma risk was concluded to be governed largely by a single major genetic variant with dominant expression that was located on an autosome and increased the risk of disease 15-fold, from a baseline risk of 5% in a Deerhound without the variant to a risk of 75% in a Deerhound that had the variant. A whole genome linkage approach was subsequently used to analyze DNA samples from 60 of these dogs and map the variant to CFA34 [6].

The conclusion that osteosarcoma risk in Deerhounds is governed largely by a single dominant genetic variant seems at odds with the conclusion reached about osteosarcoma risk in several other large and giant breeds by Karlsson et al. [7] from a genome-wide association study. When these investigators compared the DNA of affected and unaffected Greyhounds, Rottweilers, and Irish Wolfhounds, they identified multiple genetic variants associated with osteosarcoma risk in each breed, and none of the 33 genetic variants accounted for a major portion of osteosarcoma risk in any breed. Instead the associations with osteosarcoma were relatively weak with all odds ratios being <2.

The current study was undertaken to re-examine the inheritance of osteosarcoma in Scottish Deerhounds by conducting a traditional pedigree analysis on two Deerhound families, each of which was relatively highly inbred and contained many affected individuals over many generations. We believed that such an analysis could be used to test these conclusions from the 2007 study by Phillips et al. [5]:

1. Osteosarcoma risk in Scottish Deerhounds is governed primarily by a single genetic variant, which we will call a genetic risk factor in this paper,
2. The genetic risk factor lies on an autosome, and
3. The genetic risk factor has a dominant mode of inheritance.

## Methods

### Data set

Pedigrees and data on age (if alive) or age at death and osteosarcoma status for two families of Scottish Deerhounds were obtained primarily from two long-time North American Scottish Deerhound breeders whose breeding programs have produced multiple affected dogs over multiple generations. Breeder K provided information on 91 dogs representing 15 litters, which were designated as Cohort K. Breeder T provided information on 104 dogs, representing 12 litters, which were designated as Cohort T.

Where necessary, we have identified an individual dog by a code in order to keep the identities of individual dogs confidential. Males were given three-letter codes, and females were given four-letter codes.

### Assignment of phenotype

We relied on phenotype information provided by the breeders of each cohort and, in some instances, on information solicited from individual dog owners. The breeder of Cohort K is a veterinarian, and she made or confirmed the diagnosis of osteosarcoma in affected dogs in that cohort. In addition to one of the authors (JED) and the breeder of Cohort K, we spoke with four other veterinarians who are long-time Deerhound owners and/or breeders. Together, these six veterinarians have over 100 years of experience with the breed. Each confirmed that any tumor arising in a long bone of a limb of a Scottish Deerhound is overwhelmingly likely to be osteosarcoma. While this does not rule out the possibility of a misdiagnosis in a dog in our data set, it gave us confidence in using the phenotype information that we received.

We used 8.5 years old as the cutoff age at which we would assign a Deerhound the unaffected phenotype; i.e., if a dog had not developed osteosarcoma by that age, then we considered it unaffected. This cutoff age was is a compromise, which we selected after examining what each pedigree would look like using cutoff ages of 6.5, 7.5, 8.5, and 9.5 years old.

On the one hand, using a cutoff age of 8.5 years old meant that younger dogs that had not developed osteosarcoma could not be assigned to either phenotype (affected or unaffected). If these dogs were offspring in a litter, then they could contribute no useful information and so were excluded from analysis. This reduced Cohorts K from 91 to 54 usable dogs and Cohort T from 104 to 56 usable dogs. A cutoff age of 9.5 years old would have reduced the size of each cohort even further, to the point where we felt there were too few individuals for a meaningful analysis.

On the other hand, using a cutoff age of 8.5 years old risked assigning the wrong phenotype (unaffected) to some dogs that might go on to develop osteosarcoma. We felt this risk was relatively small. Based on health survey data, the average age of onset for osteosarcoma in Scottish Deerhounds is reported to be 7.2 years in males and 7.8 years in females [2]. One of us (JED) had access to the data used to calculate those averages, which are summarized in Table 1.

**Table 1:** 2011 Health Survey Data on Osteosarcoma in Scottish Deerhounds

Onset Age (years)^a^										
	>3.5 to 4.5	>4.5 to 5.5	>5.5 to 6.5	>6.5 to 7.5	>7.5 to 8.5	>8.5 to 9.5	>9.5 to 10.5	>10.5 to 11.5	>11.5	Total
No. diagnosed										
Male	2	4	4	5	1	5	1	2	0	24
Female	0	1	5	10	5	8	3	2	1	35
Cumulative total										
Male	8%	25%	42%	63%	**67%**	88%	92%	100%	---	
Female	---	3%	17%	46%	**60%**	83%	91%	97%	100%	

^a^The bolded numbers represent the likelihood that an affected Deerhound would develop osteosarcoma by the cutoff age of 8.5 years old that we used for our analysis

As the table shows, only 33% of male Deerhounds and 40% of Deerhound bitches that developed osteosarcoma were diagnosed after they reached 8.5 years old. Based on two separate health surveys [2, 3], the lifetime incidence of osteosarcoma in Deerhounds averages about 9% in males and 11% in bitches. Consequently, the chances of mistakenly designating a dog over 8.5 years old as unaffected were only 3% for males (33% × 9%) and 4% for bitches (40% × 11%).

### Analysis method

To analyze the pedigree and health information, we began with the hypothesis that osteosarcoma risk in each Scottish Deerhound cohort was governed largely by a single genetic risk factor and then made the following assumptions:

1. The genetic risk factor was highly penetrant; i.e., expression of the osteosarcoma phenotype was not greatly influenced by other genetic or environmental factors, so that if a Deerhound inherited the risk factor (one or two copies, depending upon whether the risk factor is dominant or recessive) and lived long enough, then it was very likely to develop osteosarcoma.
2. Osteosarcoma rarely occurred in Deerhounds that did not carry the genetic risk factor, so that if a dog lived long enough and did not develop osteosarcoma, then it very likely either did not carry the genetic risk factor (if the factor was dominant) or carried only a single copy (if the factor was recessive).
3. Osteosarcoma in Deerhounds represented a single phenotype; i.e., there were not multiple types of osteosarcoma governed by different genetic risk factors. As a corollary to this assumption, if the genetic risk factor for osteosarcoma had dominant expression, then the osteosarcoma phenotype would be the same whether the affected individual was heterozygous or homozygous.

We analyzed the information from each cohort separately, rather than assuming that the same genetic risk factor was present in both families. This was a conservative approach. Because Scottish Deerhounds have always been a rare breed with a small breeding population, a good deal of close breeding has occurred. Therefore, it is reasonable to assume that, if a single genetic factor was governing osteosarcoma risk in Deerhounds, then it would be the same factor throughout the breed. On the other hand, there was no overlap (i.e., no dogs in common) in the pedigrees for the two cohorts going back at least 7 generations, and therefore no a priori reason to assume that the same risk factor was operating in both cohorts.

#### Assuming recessive expression

When we assumed recessive expression, we used a lower-case letter “t” (for tumor) to designate the osteosarcoma risk factor and an upper case “T” to represent the dominant normal sequence, and we also assumed the risk factor had 100% penetrance. These premises followed:

R1. An affected dog was always genotype t/t.

R2. An unaffected dog was always either genotype T/t or genotype T/T. If it was genotype T/t, then it was a silent carrier of the risk factor.

R3. If an unaffected parent produced an affected pup, then the parent must be a silent carrier (genotype T/t).

R4. If an affected parent was crossed with an unaffected parent, and the resulting litter contained both affected and unaffected pups, then each unaffected pup must be a silent carrier (genotype T/t).

#### Assuming dominant expression

When we assumed dominant expression, we used an upper-case letter “C” (for cancer) to designate the osteosarcoma risk factor and a lower case “c” to represent the recessive normal sequence. Unlike when analyzing for recessive expression, we did not assume 100% penetrance. This was because an initial review of the data showed that each cohort contained at least one cross in which two unaffected parents had produced an affected pup, which would be unlikely if the risk factor were dominant and 100% penetrant. (Such a result could happen if we had mistakenly designated a parent as unaffected, but the chance of this was ≤4%, as previously discussed).

For a population being studied, penetrance is defined as the proportion of individuals with a particular genotype that also express an associated phenotype. Thus, penetrance is impossible to calculate unless one knows the genotype of individuals. Penetrance is said to be incomplete when some individuals with the disease-causing genotype fail to develop the disease. If a genetic risk factor is dominantly expressed but has a relatively low penetrance, then many individuals that carry the risk factor will not develop the disease, and disease risk will appear to be random rather than heritable [8]. Osteosarcoma risk in our two Deerhound cohorts does not fit that description; instead, risk appears clearly heritable in each cohort, as was previously reported for Deerhounds [5]. Consequently, we ruled out the possibility that osteosarcoma risk was dominant with low penetrance and instead analyzed for the possibility of dominant expression with high (but incomplete) penetrance. Incomplete penetrance can result from the influence of other environmental or genetic factors. In the latter case, saying that a risk factor has incomplete penetrance is simply another way of saying that at least one other risk factor influences the phenotype. For a late-onset disease like osteosarcoma, penetrance also can appear to be incomplete because a dog with the genetic risk factor simply died of some other cause before living long enough for osteosarcoma to arise. As already described, we reduced this possibility greatly (probably to ≤4%) by using a cutoff age of 8.5 years old to designate a dog as unaffected.

Unlike with recessive expression, a dog’s phenotype does not reveal its full genotype when one assumes dominant expression. Nevertheless, if we assumed a single dominant risk factor with high (but incomplete) penetrance, then these premises followed:

D1. Affected dogs were always either genotype C/c or C/C.

D2. Unaffected dogs could be any genotype (C/C, C/c, or c/c). If they are C/C or C/c, then they are silent carriers of the risk factor.

D3. According to the definition of high penetrance, most dogs that carried the risk factor would develop osteosarcoma, and silent carriers would be uncommon. In other words, most unaffected dogs would be genotype c/c, and very few would be genotype C/C or C/c.

D4. If a cross between two unaffected dogs produced an affected pup, then one or both of the parents was a silent carrier.

D5. Because silent carriers would be uncommon, crosses between two silent carriers would be very uncommon. Instead, when two unaffected dogs were crossed, it would be very likely that both were genotype c/c, unlikely (but still possible) that one was a silent carrier (genotype C/C or C/c), and very unlikely that both were silent carriers.

D6. For any cross where one parent was affected and the other was not, there would be two possible genotypes for the affected parent (C/C or C/c) and three possible genotypes for the unaffected parent (C/C, C/c, or c/c). In keeping with Premise D3, the unaffected parent would be much more likely to be genotype c/c than a silent carrier. Table 2 summarizes the possible parental genotypes, the resulting average pup phenotype distribution for each cross, and the likelihood of each result.

**Table 2:** Possible Pup Phenotypes for Litters Produced by an Affected Dam and an Unaffected Sire Assuming Dominant Expression

Possible genotypes		Expected phenotypes in pups		Likelihood^a^
Unaffected Sire	Affected Dam	Affected	Unaffected	
c/c	C/c	50%	50%	High
	C/C	100%	0%	
C/c	C/c	75%	25%	Low
	C/C	100%	0%	
CC	C/c	100%	0%	
	C/C	100%	0%	

^a^Based on the likelihood of the sire’s genotype

### Pedigree/phenotype/genotype diagrams

The diagrams in this paper contain only parents and evaluable offspring; i.e., they exclude offspring that died osteosarcoma-free before 8.5 years of age. For phenotype, males are represented by squares, and females are represented by circles. An empty symbol means the dog was unaffected (osteosarcoma-free at death), while a filled symbol means the dog developed osteosarcoma. If the phenotype has been inferred, rather than being provided by the breeder, then the symbol contains a dot. For Cohort K, we also show genotype based on assuming a single recessive risk factor, using a lowercase “t” for the recessive risk factor and an uppercase “T” for the dominant normal counterpart.

## Results

**Fig. 1:**
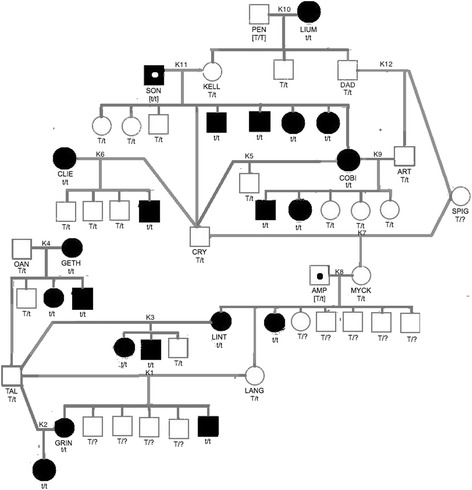
Pedigree for Cohort K, Including Genotypes Based on the Assumption of a Recessive Risk Factor. Legend: Square = male, circle = female, filled = affected, empty = unaffected, filled with dot = phenotype unknown but inferred from the dog’s genotype

**Fig. 2:**
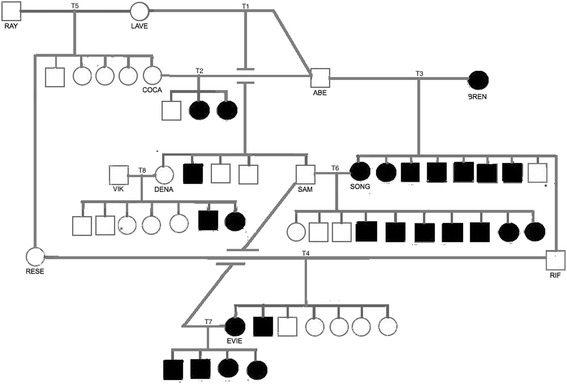
Pedigree for Cohort T. Legend: Square = male, circle = female, filled = affected, empty = unaffected

Cohort K (Fig. 1) contained ten litters for which the phenotypes of both parents were known (K1-K7 and K9-K10) and two other litters for which the phenotype of only one parent was known (K8 and K11). Cohort T (Fig. 2) contained six litters for which the phenotypes of both parents were known (T1-T3, T5-T7) and two litters for which the phenotype of only one parent was known (T4 and T8).

A brief glance reveals that the osteosarcoma phenotype seems clearly heritable in both cohorts. But differences emerge if one examines the litters that were produced when an affected dog was bred to an unaffected dog. Cohort K contained seven such crosses (Litters K2-K6, K9, and K10), which resulted in the production of 8 affected pups and 13 unaffected pups, while Cohort T contained three such crosses (Litters T3, T6, and T7), which resulted in the production of 18 affected pups and 5 unaffected pups. In other words, having a parent that developed osteosarcoma raised a pup’s risk of developing osteosarcoma to 38% in Cohort K but to 78% in Cohort T.

Possible explanations for this difference include:

1. Osteosarcoma risk was governed by a different single genetic risk factor in each cohort, and each risk factor had a different mode of expression; i.e., the risk factor in Cohort K was recessive, while the risk factor in Cohort T was dominant.
2. Osteosarcoma risk was governed by the same single genetic risk factor in each cohort, but the risk factor was more prevalent in Cohort T than Cohort K. For a recessive risk factor, this would mean that an unaffected dog would be much more likely to be a heterozygous carrier than to lack the risk factor. For a dominant risk factor, this would mean that an affected dog would be much more likely to be homozygous than heterozygous for the risk factor.
3. Osteosarcoma risk was governed by multiple genetic risk factors in each cohort, and one or more of the risk factors had become fixed in Cohort T.

To distinguish among these possible explanations, we analyzed the data from each cohort in two ways, first by asking how well the results could be explained by a single recessive risk factor and next by asking how well the results could be explained by a single dominant risk factor.

### Cohort K

#### How well could osteosarcoma inheritance in Cohort K be explained by assuming a single recessive risk factor (t)?

To evaluate this possibility, we examined litters produced by two unaffected parents and litters where one parent was affected and the other unaffected.

##### Both parents unaffected

Litters K7 and K12 contained only a single unaffected pup, and so were not helpful. But Litter K1 contained two affected pups and four unaffected pups. The sire and dam of Litter K1 (TAL and LANG) were osteosarcoma-free when they died at 10 and 12 years of age, respectively. The production of an affected pup by two unaffected parents is a hallmark of recessive expression, although it does not rule out dominant expression.

If we assumed that a single recessive genetic factor governed osteosarcoma risk in Cohort K, then the following must be true:

- Both TAL and LANG were heterozygous carriers (genotype T/t). This is certainly possible, as each belonged to a litter with affected siblings.
- The osteosarcoma risk factor resides on an autosome and not on the X chromosome, because otherwise there could be no male heterozygous carrier such as TAL.
- Based on Premise R3, the sires for litters K4 (OAN), K6 (CRY), and K9 (ART) must be heterozygous carriers (genotype T/t).
- Based on Premise R4, the 14 unaffected pups in Litters K3-K6, K9, and K10 must be heterozygous carriers (genotype T/t).

Based on these considerations, we assigned putative genotypes to many of the dogs in Cohort K, as shown in Fig. 1. There were, however, several dogs for which we could not clearly infer a genotype.

SON sired Litter K11 by dam KELL and died at 6 years old without developing osteosarcoma. The litter contained five affected pups, so both parents carried the osteosarcoma risk factor. KELL died osteosarcoma-free at more than 9 years old, and so was likely a heterozygous carrier. Litter K11 also contained four unaffected pups. The approximately 1-to1 ratio of affected to unaffected pups suggests (but does not prove) that SON was homozygous for the risk factor, rather than a heterozygous carrier, and would have developed osteosarcoma if he had lived long enough. If that is true, then the four unaffected pups in Litter K11 were heterozygous carriers. We already had deduced this to be true for one of those pups, CRY, based on the occurrence of an affected pup in Litter K6 that she produced.

PEN sired Litter K10 by dam LIUM. He died osteosarcoma-free at 12 years old, but LIUM died of osteosarcoma when she was 10 years old. Litter K10 consisted of three unaffected pups. Two more unaffected pups lived to be more than 8 years old (data not shown). This suggests (but does not prove) that PEN was genotype T/T; i.e., a homozygous unaffected dog.

SPIG produced Litter K7 by sire CRY, which we had deduced to be a heterozygous carrier. SPIG was osteosarcoma-free when she died at 13 years old. Because Litter K7 contained only a single unaffected pup, we cannot deduce anything about SPIG’s genotype.

Sire AMP and dam MYCK produced Litter K8. AMP died osteosarcoma-free at 6 years old and so could not be assigned a phenotype, but dam MYCK died osteosarcoma-free at 9.5 years old and so was considered to be unaffected. Litter K8 contained two affected pups and six unaffected pups, one of which was LANG, which we have deduced to be a heterozygous carrier, based on the occurrence of affected pups in Litter K1 that she produced. The occurrence of affected pups in Litter K8 means that both parents carried the osteosarcoma risk factor. Therefore, MYCK was a heterozygous carrier. The 1-to-4 ratio of affected to unaffected offspring in Litter K8 strongly suggests (but does not prove) that AMP was homozygous for the risk factor, rather than a heterozygous carrier, and would have developed osteosarcoma if he had lived long enough.

##### One parent affected and the other unaffected

Once we had inferred potential genotypes as described, we re-examined the six litters in which one parent was affected and the other was deduced to be a heterozygous carrier (Litters K2-K6 and K9). If the assumption about a single recessive genetic risk factor were correct, then such a cross should produce, on average, a 1-to-1 ratio of affected to unaffected pups. The ratios of affected to unaffected pups were 1-to-0, 2-to-1, 2-to-2, 0-to-1, 1-to-3, and 2-to-3 for Litters K2-K6 and K9, respectively, for an overall ratio of 8-to-10, or 0.8-to-1.

There are several factors that might account for the slightly lower-than-expected ratio of affected to unaffected pups in these six litters, such as:

- Chance, reflecting the relatively small sample size.
- A single pup in one of the litters was mistakenly assigned to the unaffected phenotype. This could happen if the pup had died osteosarcoma-free after reaching 8.5 years old but would have developed osteosarcoma if it had lived long enough.
- Osteosarcoma risk in Cohort K was not governed solely by a single recessive risk factor but instead was influenced also by an environmental factor or other genetic factor(s).

#### How well could osteosarcoma inheritance in Cohort K be explained by assuming a single dominant risk factor (C)?

To evaluate this possibility, we examined litters produced by two unaffected parents and litters where one parent was affected and the other unaffected.

##### Both parents unaffected

Litter K1, produced by two unaffected parents, contained two affected pups. While this result is typical of a recessive risk factor, it also can occur if a dominant risk factor has incomplete penetrance (<100% expression). Thus, the results for Litter K1 do not rule out the possibility of a dominant risk factor with relatively high penetrance.

##### One parent affected and the other unaffected

Cohort K contained seven litters produced by affected dams and unaffected sires. Based on Premise D6, one would expect the incidence of affected pups to average 75%. Table 3 shows the actual phenotypic compositions of Litters K2-K6, K9, and K10.

**Table 3:** Pup Phenotypes for Cohort K Litters Produced by an Affected Dam and an Unaffected Sire

Litter	Absolute phenotypes		Relative phenotypes	
	Affected	Unaffected	Affected	Unaffected
K2	1	0	100%	0%
K3	2	1	67%	33%
K4	2	2	50%	50%
K5	0	1	0%	100%
K6	1	3	25%	75%
K9	2	3	40%	60%
K10	0	3	0%	100%
Totals/Means	8	13	38%	62%

The ratio of affected to unaffected pups was 8-to-13, or 0.62-to-1. This is less than the lowest ratio one would expect, which is 1-to-1 if each sire was genotype c/c and each dam was heterozygous (genotype C/c). One possible explanation for the lower-than-expected result is that the risk factor was not dominant. But there are other possible explanations, not mutually exclusive, such as:

- Chance, reflecting the relatively small sample size.
- Two pups were mistakenly assigned to the unaffected phenotype. This could happen if they had died osteosarcoma-free after reaching 8.5 years old but would have developed osteosarcoma if they had lived long enough.
- The dominant risk factor had relatively low penetrance; i.e., osteosarcoma risk in Cohort K was not governed solely by a single dominant risk factor but instead was influenced also by an environmental factor or other genetic factor(s).

### Cohort T

#### How well could osteosarcoma inheritance in Cohort T be explained by assuming a single recessive risk factor (t)?

As a first step, we examined the three litters in Cohort T for which both parents were unaffected. Litter T5 contained six unaffected pups, and so was not helpful. But Litters T1, T2, and T8 each contained affected pups. The production of affected pups by two unaffected parents is the hallmark of recessive expression.

If a single recessive genetic factor governed osteosarcoma risk in Cohort T, then the following must be true:

- The five unaffected parents that produced Litters T1, T2, and T8 (ABE, LAVE, COCA, VIK, and DENA) were all heterozygous carriers (genotype T/t).
- The osteosarcoma risk factor resides on an autosome and not on the X chromosome, because otherwise there could be no male heterozygous carriers such as ABE and VIK.
- Based on Premise R3, the sire of Litter T6 (SAM) must be a heterozygous carrier (genotype T/t).
- Based on Premise R4, the 5 unaffected pups in Litters T3 and T6 (including RIF, the sire of Litter T4) must be heterozygous carriers (genotype T/t).

Once we had inferred potential genotypes as described, we re-examined the three litters in which one parent was affected (genotype t/t) and the other was deduced to be a heterozygous carrier (genotype T/t). If the assumption about a single recessive genetic risk factor were correct, then such a cross should produce, on average, a 1-to-1 ratio of affected to unaffected pups. In Cohort T, the ratios of affected to unaffected pups were 7-to-2, 7-to-3, and 4-to-0 for Litters T3, T6, and T7, respectively, for an overall ratio of 18-to-5 or approximately 4-to-1. This is so unlikely to occur with a recessive risk factor that it makes that hypothesis highly unlikely for Cohort T.

#### How well could osteosarcoma inheritance in Cohort T be explained by assuming a single dominant risk factor (C)?

To evaluate this possibility, we examined litters produced by two unaffected parents and litters where one parent was affected and the other unaffected.

##### Both parents unaffected

Cohort T contained four litters for which both parents were unaffected. For such litters, there were three possible genotypes for each parent (C/C, C/c, or c/c); however, in keeping with Premise D3, each parent was much more likely to be genotype c/c than to be a silent carrier of genotype C/C or C/c. Because of this, the most likely outcome of a cross between unaffected parents would be 0% affected pups. In the unlikely event that one parent was a silent carrier, one would expect either 50% or 100% affected pups, depending upon whether the carrier parent was genotype C/c or C/C, respectively. In the most unlikely event that both parents were silent carriers, one would expect either 75% or 100% affected pups, depending upon whether both parents were genotype C/c or one was genotype C/C, respectively.

Litter T5 consisted of six unaffected pups, which was the most likely outcome. But Litters T1, T2, and T8 each contained an affected pup, which would be possible only if each litter had at least one parent that was a silent carrier of the dominant risk factor.

For Litters T1, T2, and T8, the ratios of affected to unaffected pups were 1-to-3, 2-to-3, and 2-to-5, respectively, for an overall ratio of 5-to-11, or 0.45-to-1. This is less than the lowest ratio one would expect, which is 1-to-1 if each sire was genotype c/c and each dam was heterozygous (genotype C/c). One possible explanation for the lower-than-expected result is that the risk factor was not dominant. But there are other possible explanations, not mutually exclusive, such as:

- Chance, reflecting the relatively small sample size.
- Three pups were mistakenly assigned to the unaffected phenotype. This could happen if they had died osteosarcoma-free after reaching 8.5 years old but would have developed osteosarcoma if they had lived long enough.
- The dominant risk factor had relatively low penetrance; i.e., osteosarcoma risk in Cohort T was not governed solely by a single dominant risk factor but instead was influenced also by an environmental factor or other genetic factor(s).

##### One parent affected and the other unaffected

Cohort T contained three litters produced by affected dams and unaffected sires. Based on Premise D6, one would expect the incidence of affected pups to average 75%. Table 4 shows the phenotypic compositions of Litters T3, T6, and T7.

**Table 4:** Pup Phenotypes for Cohort T Litters Produced by an Affected Dam and an Unaffected Sire

Litter	Absolute phenotypes		Relative phenotypes	
	Affected	Unaffected	Affected	Unaffected
T3	7	2	78%	22%
T6	7	3	70%	30%
T7	4	0	100%	0%
Totals/Means	18	5	78%	22%

The 78% incidence of osteosarcoma is very close to the 75% average incidence that one would expect with a dominant risk factor that was completely penetrant.

## Discussion

When it comes to analyzing inheritance patterns for diseases that affect Scottish Deerhounds, osteosarcoma offers some advantages but also presents some challenges. One advantage is that the disease is relatively common. The Scottish Deerhound Club of America conducted health surveys in 1996 and 2011 that gathered responses from Deerhound owners and breeders. The 2011 survey yielded information on 588 dogs (273 males and 315 bitches), of which 24 males and 35 bitches had developed osteosarcoma [2]. These numbers translate into incidences of 9 and 11%, respectively.

One must be careful not to over-interpret these incidence numbers. On the one hand, any survey that is not randomly conducted tends to overestimate the incidence of a health problem because people whose dogs have experienced a problem are more likely to participate in the survey than people whose dogs are healthy. The health survey conducted by the Scottish Deerhound Club of America did not randomly sample the Deerhound population, and so it is possible that the true incidence of osteosarcoma is lower than the numbers indicate.

On the other hand, because osteosarcoma arises later in life, there is also a bias in the other direction, which arises because the survey results included many dogs that were still alive and too young to have had a chance to develop osteosarcoma. Some of those dogs will go on to develop osteosarcoma. Thus, the survey will have tended to underestimate the true incidence of osteosarcoma in Deerhounds.

One way to get some idea of how much bias was introduced by including dogs that were still alive is to analyze the data only from those Deerhounds which had died. For the 2011 survey, the appropriate subpopulations were bitches born before January 10, 1999 and males born before July 20, 2000, because all of them had died. These subpopulations consisted of 118 bitches and 93 males. Table 5 compares the incidence of osteosarcoma in the entire survey population and these subpopulations.

**Table 5:** Incidence of Osteosarcoma in 2011 Survey

	Males	Bitches
Entire population^a^	9%	11%
Subpopulation^b^	12%	16%

^a^Entire population is 273 males and 317 bitches

^b^Subpopulation is 93 males born before 7/20/2000 and 118 bitches born before 1/10/1999, all of which have died

This analysis suggests that the bias introduced by including data from all dogs (alive and dead) in calculating the incidence of osteosarcoma is not trivial. However, there also is a bias in the other direction because the survey was not randomly conducted. It is impossible to know how these two biases—one that favors overestimation and other that favors underestimation—played out, but it seems reasonable to expect that the biases canceled each other out and that the true incidence of osteosarcoma in Scottish Deerhounds is approximately that shown for the entire population in Table 5.

When analyzing inheritance patterns, osteosarcoma offers another advantage over other diseases: the affected phenotype is easy to identify without any advanced diagnostic testing. Any tumor that arises in the long bone of the limb of a middle-aged or old Scottish Deerhound can be presumed to be osteosarcoma.

The main challenge of investigating osteosarcoma in Scottish Deerhounds is that the disease arises late in life. In the 2011 health survey conducted by the Scottish Deerhound Club of America, the age of onset ranged from 5 to 11 years old in bitches, with a mean of 7.8 years old and a median of 8 years old. In males, the age of onset ranged from 4 to 10 years old, with a mean of 7.2 years old and a median of 7 years old.

As a consequence of the late onset of osteosarcoma, one has to make assumptions about how old a Deerhound should be in order to be considered unaffected. For this study, we chose the age of 8.5 years old as our cutoff point, assigning the “unaffected” phenotype only to a dog that was osteosarcoma-free and at least 8.5 years old.

For Cohort K, the inheritance of osteosarcoma could be explained reasonably well if we assumed that risk was governed by a single recessive genetic factor. When we made that assumption and then deduced genotypes where possible, there were six litters in which one parent was affected and the other was deduced to be a heterozygous carrier (Litters K2-K6 and K9). Together, those litters contained 8 affected and 10 unaffected pups, which is very close to the 1-to-1 ratio that one would expect if the assumption about a single recessive genetic risk factor were correct. In addition, a cross between two unaffected parents in Cohort K produced a litter with two affected pups, which is one of the hallmarks of a recessive genetic risk factor.

On the other hand, although production of affected offspring by two unaffected parents is typical of a recessive risk factor, the same result can occur with a dominant risk factor that has incomplete penetrance. Thus, the production of affected pups by unaffected parents in Cohort K does not rule out the possibility of a dominant risk factor, if that factor is incompletely penetrant. When we assumed that osteosarcoma risk was governed by a single dominant genetic factor and evaluated the seven litters in Cohort K that were produced by an affected dam and unaffected sire, we found that those litters contained 8 affected and 13 unaffected pups, for a ratio of 0.62-to-1. This is less than the lowest ratio one would expect from such crosses, which is 1-to-1 if each dam was a heterozygous carrier. But this result does not mean that risk is not governed by a dominant genetic factor. Instead, the lower-than-expected ratio could be due to chance (reflecting the relatively small sample size), mistaken assignment of two pups to the unaffected phenotype, or relatively low penetrance of the risk factor.

For Cohort T, the inheritance of osteosarcoma was inconsistent with the assumption that risk is governed by a single recessive genetic factor. If we assumed a single recessive risk factor and then deduced genotypes where possible, there were three litters in which one parent was affected and the other was deduced to be a heterozygous carrier (Litters T3, T6, and T7). Together, those litters contained 18 affected and 5 unaffected pups, which is very different from the 1-to-1 ratio that one would expect if the assumption about a single recessive genetic risk factor were correct. On the other hand, the 18-to-5 ratio of affected to unaffected pups is almost exactly what one would expect if osteosarcoma risk were governed by a single dominant risk factor with high penetrance.

On balance, our analysis suggests that osteosarcoma risk in Cohort K was likely to have been governed by a single recessive genetic factor. However, we cannot rule out the possibility that risk was governed instead by a dominant genetic factor with relatively low penetrance; i.e., that risk was influenced by one or more other environmental or genetic factors. The idea that osteosarcoma risk is influenced by multiple genetic factors rather than a single factor is more in keeping with current thinking [9].

In contrast, our analysis strongly suggests that osteosarcoma risk in Cohort T was governed largely by a single dominant risk factor with high penetrance, as has been reported previously for Deerhounds [5].

We were initially surprised that our analysis suggested a different genetic risk factor for osteosarcoma might be operating in the two Deerhound families we studied. The breeding population of Deerhounds has always been small, and the application of Occam’s Razor would lead one to expect that a single genetic risk factor was driving osteosarcoma risk in all members of the breed.

In retrospect, our results in Deerhounds may not be so surprising in light of a recent genome-wide association study (GWAS) [7] that identified multiple genetic markers for osteosarcoma in three other breeds with a high incidence of the disease—14 in Greyhounds, 15 in Rottweilers, and 4 in Irish Wolfhounds. The osteosarcoma markers were different in each breed with no overlap; i.e., the investigators identified 33 different genetic markers for osteosarcoma in only three breeds.

There are two reasons why a GWAS might identify a genetic marker for osteosarcoma in one breed but not in another: the genetic marker might really be absent in the second breed or it might instead have become fixed in the second breed and so have become invisible by GWAS. To test for fixed markers, Karlsson et al. screened each breed for all 33 different genetic markers. They found 8 markers that had become fixed in one other breed and a single genetic marker that had become fixed in both other breeds, which was a marker on chromosome 11. This marker was present in more affected Greyhounds (87%) than unaffected Greyhounds (68%), but it was present in essentially all Irish Wolfhounds (95%) and Rottweilers (97%), regardless of osteosarcoma status.

Karlsson et al. next checked six other osteosarcoma-prone breeds for the marker on chromosome 11. In 73 Golden Retrievers, the pattern matched that in Rottweilers and Irish Wolfhounds, with the marker being fixed and present in essentially all dogs, whether affected (95%) or not (92%). In 55 Leonbergers and 37 Great Pyrenees, the pattern matched that in Greyhounds, with the marker being present in most individuals but somewhat more common in affected than unaffected dogs. In Leonbergers, the marker was present in 77% of affected dogs and 62% of unaffected dogs, while in Great Pyrenees, the marker was present in 78% of affected dogs and 62% of unaffected dogs. In Mastiffs, Great Danes, and Labrador Retrievers, the marker on chromosome 11 was relatively uncommon. In Great Danes, it was present at a similar low frequency in affected and unaffected dogs, but in Mastiffs and Labrador Retrievers, it was present in more affected dogs than unaffected dogs.

The discovery that genetic markers for osteosarcoma have become fixed in several breeds at high risk for the disease suggests a possible explanation for our results. If a single dominant genetic factor was governing much of the osteosarcoma risk in both Deerhound cohorts that we studied, but one or more other genetic risk factors had become fixed in Cohort T but not in Cohort K, then the dominant risk factor would appear to be more highly penetrant in Cohort T than Cohort K.

There is an important difference between the results reported for Deerhounds in Cohort T in our study and for a large Deerhound population in an earlier study by Phillips et al. [5] and the results reported for Greyhounds, Rottweilers, and Irish Wolfhounds by Karlsson et al. [7]. In Deerhounds, our analysis of Cohort T and the analysis by Phillips et al. suggest that osteosarcoma risk is governed largely by a single genetic variant that lies on an autosome; i.e., a single genetic variant is rather strongly associated with the osteosarcoma phenotype, and the nature of the association is cause and effect. But in Greyhounds, Rottweilers, and Irish Wolfhounds, no single genetic variant was strongly associated with osteosarcoma in any breed. Instead, the GWAS identified multiple genetic variants in each breed that were only weakly associated with osteosarcoma; specifically:

- The highest odds ratio (OR) in Irish Wolfhounds was for SNP BICF2P1125643 (OR = 1.75), which was present in only 4 of 28 affected dogs (14%).
- The highest OR in Rottweilers was for SNP BICF2P411325 (OR = 1.43), which was absent in only 27% of unaffected dogs.
- The highest ORs in Greyhounds were for SNP BICF2P133066 and SNP TIGRP21P215623 (each OR = 1.36). The first was present in only 12% of affected dogs, while the latter was absent in only 11% of unaffected dogs.

Possibly Karlsson et al. did not find a SNP that was strongly associated with osteosarcoma in any of the breeds they studied because no single genetic variant governs osteosarcoma risk in those breeds. We think this is unlikely because in at least one family of Irish Wolfhounds, osteosarcoma risk is reportedly governed largely by a single genetic variant with an autosomal inheritance pattern [10], as appears also to be true in at least some Deerhound families.

In fact, the results obtained by Karlsson et al. are in keeping with those of many other GWASs. For reasons that are still unclear, GWASs routinely fail to identify a genetic variant strongly associated with a disease phenotype [11]. There has been much speculation about why this is so [12, 13].

The previous analysis by Phillips et al. [5] suggested that the inheritance of osteosarcoma in Scottish Deerhounds was governed largely by a single genetic variant with dominant expression. We concluded the same thing for our Cohort T. However, our analysis suggested that osteosarcoma in Cohort K was governed either largely by a single genetic variant with recessive expression or by a combination of an incompletely dominant genetic factor and at least one other genetic or environmental factor. When we checked with the breeders who supplied us with information on each cohort, we learned that many individuals in Cohort T had also been included in the population analyzed by Phillips et al., but that Cohort K had not been part of that population. This may explain why our results for Cohort T matched those of Phillips et al.

The analysis described in this paper will be followed by genetic mapping. For both of the Deerhound families that we studied, DNA samples from at least some of the deceased dogs are banked in one or more repositories. With the help of the Scottish Deerhound Club of America, we are currently arranging for access to those samples, and also arranging to obtain DNA samples from dogs in both families that are still alive, and from their descendants. Our initial intention is to screen the samples for osteosarcoma-related genetic variants previously identified in Deerhounds, Wolfhounds, Greyhounds, and Rottweilers.

## Conclusions

Inheritance of osteosarcoma in two Scottish Deerhound families could be explained well by a single genetic risk factor residing on an autosome, consistent with a previous report. In one family, inheritance was consistent with dominant expression, as previously reported. In the other family, inheritance fit better with recessive expression, although the possibility of a dominant genetic factor influenced by one or more other genetic factors could not be ruled out. In either case, the results suggest that there may be at least two different genetic risk factors for osteosarcoma in Deerhounds.

## References

[1] Tjalma RA (1966). Canine bone sarcoma: estimation of relative risk as a function of body size. J Natl Cancer Institute.

[2] Dillberger JE. 2011 Deerhound health survey results – part 2. The Claymore. Newsletter of the Scottish Deerhound Club of America 2012; May/June: 9–13.

[3] Dillberger JE. Deerhound health survey. Part 2. Health problems. The Claymore. Newsletter of the Scottish Deerhound Club of America 1996; May/June: 21–40.

[4] Dillberger JE. 2011 Deerhound health survey results – part 1. The Claymore. Newsletter of the Scottish Deerhound Club of America 2012; March/April: 15–18 and 27–28.

[5] Phillips JC, Stephenson B, Hauck M, Dillberger J (2007). Heritability and segregation analysis of osteosarcoma in the Scottish Deerhound. Genomics.

[6] Phillips JC, Lembcke L, Chamberlin T (2010). A novel locus for canine osteosarcoma (OSA1) maps to CFA34, the canine orthologue of human 3q26. Genomics.

[7] Karlsson EK, Sigurdsson S, Ivansson E, Thomas R, Elvers I, Wright J, Howald C, Tonomura N, Perloski M, Swofford R, Biagi T, Fryc S, Anderson N, Courtay-Cahen C, Youell L, Ricketts SL, Mandlebaum S, Rivera P, von Euler H, Kisseberth WC, London CA, Lander ES, Couto G, Comstock K, Starkey MP, Modiano JF, Breen M, Lindblad-Toh K. Genome-wide analyses implicate 33 loci in heritable dog osteosarcoma, including regulatory variants near CDKN2A/B. Genome Biology 2013; 14: R132 at http://genomebiology.com/2013/14/12/R132. Accessed 21 Feb 2017. 10.1186/gb-2013-14-12-r132 PMC4053774 24330828

[8] Griffiths AJF, Gelbart WM, Lewontin RC, Miller JH (2002). Modern genetic analysis. Integrating genes and genomes.

[9] Griffin G, Urfer SR (2011). Inherited osteosarcoma in a family of Irish Wolfhounds. Conference: Proceedings of the Third Canine Science Forum, At Barcelona, Volume: CSF 12. DOI: 10.13140/RG.2.1.3924.5680. At https://www.researchgate.net/publication/301552608_Inherited_Osteosarcoma_in_a_Family_of_Irish_Wolfhounds. Accessed 21 Feb 2017.

[10] Fenger JM, London CA, Kisseberth WC (2014). Canine osteosarcoma: a naturally occurring disease to inform pediatric oncology. Institute for Lab Anim Res J.

[11] Alvarez CE (2014). Naturally occurring cancers in dogs: insights for translational genetics and medicine. Institute for Lab Anim Res J.

[12] Ku CS, Loy EY, Pawitan Y, Chia KS (2010). The pursuit of genome-wide association studies: where are we now?. J Human Genetics.

[13] Manolio TA (2010). Genomewide association studies and assessment of the risk of disease. N Engl J Med.

